# Supplementary material for: Modeling glioblastoma heterogeneity as a dynamic network of cell states
Source: Mol Syst Biol. 2021 Sep 16;17(9):e10105. doi: 10.15252/msb.202010105 (PMC8444284; doi:10.15252/msb.202010105)
Supplement: Supplementary file 5 — Source Data for Figure 3 [file MSB-17-e10105-s001.zip › Figure3A_sourcedata/GSEA_3065/nosigdb_state5.GseaPreranked.1622718435914/GAROFANO_IAVARONE_NEU_SUBTYPE.html]

Details for gene set GAROFANO\_IAVARONE\_NEU\_SUBTYPE[GSEA]

|  || Dataset | state5 |
| Phenotype | NoPhenotypeAvailable |
| Upregulated in class | na\_pos |
| GeneSet | GAROFANO\_IAVARONE\_NEU\_SUBTYPE |
| Enrichment Score (ES) | 0.4604959 |
| Normalized Enrichment Score (NES) | 1.9785757 |
| Nominal p-value | 0.0 |
| FDR q-value | 0.0019750404 |
| FWER p-Value | 0.011 |
Table: GSEA Results Summary

  

Fig 1: Enrichment plot: GAROFANO\_IAVARONE\_NEU\_SUBTYPE      
 Profile of the Running ES Score & Positions of GeneSet Members on the Rank Ordered List

  

| PROBE | GENE SYMBOL | GENE\_TITLE | RANK IN GENE LIST | RANK METRIC SCORE | RUNNING ES | CORE ENRICHMENT || 1 | WSB1 |  |  | 13 | 0.861 | 0.0320 | Yes |
| 2 | BCAN |  |  | 15 | 0.850 | 0.0649 | Yes |
| 3 | SOX6 |  |  | 28 | 0.754 | 0.0928 | Yes |
| 4 | GRIA2 |  |  | 32 | 0.732 | 0.1209 | Yes |
| 5 | NKTR |  |  | 42 | 0.700 | 0.1470 | Yes |
| 6 | NCAM1 |  |  | 70 | 0.581 | 0.1666 | Yes |
| 7 | RTN3 |  |  | 123 | 0.505 | 0.1803 | Yes |
| 8 | LSAMP |  |  | 132 | 0.497 | 0.1987 | Yes |
| 9 | DPP6 |  |  | 161 | 0.475 | 0.2139 | Yes |
| 10 | CHD7 |  |  | 312 | 0.376 | 0.2115 | Yes |
| 11 | KIDINS220 |  |  | 325 | 0.372 | 0.2246 | Yes |
| 12 | GPC2 |  |  | 367 | 0.354 | 0.2337 | Yes |
| 13 | GRIN2B |  |  | 431 | 0.330 | 0.2394 | Yes |
| 14 | SRGAP3 |  |  | 446 | 0.326 | 0.2505 | Yes |
| 15 | PODXL2 |  |  | 464 | 0.321 | 0.2610 | Yes |
| 16 | NAV2 |  |  | 478 | 0.318 | 0.2719 | Yes |
| 17 | ITM2C |  |  | 551 | 0.297 | 0.2753 | Yes |
| 18 | ZEB2 |  |  | 670 | 0.271 | 0.2724 | Yes |
| 19 | DNER |  |  | 671 | 0.270 | 0.2829 | Yes |
| 20 | ZMYM2 |  |  | 673 | 0.270 | 0.2933 | Yes |
| 21 | PAG1 |  |  | 713 | 0.259 | 0.2989 | Yes |
| 22 | CADM2 |  |  | 739 | 0.253 | 0.3059 | Yes |
| 23 | RUFY3 |  |  | 839 | 0.234 | 0.3038 | Yes |
| 24 | PLXNB1 |  |  | 856 | 0.232 | 0.3110 | Yes |
| 25 | PTBP2 |  |  | 914 | 0.222 | 0.3131 | Yes |
| 26 | NXPH1 |  |  | 990 | 0.212 | 0.3129 | Yes |
| 27 | SENP7 |  |  | 994 | 0.212 | 0.3208 | Yes |
| 28 | FHIT |  |  | 1042 | 0.206 | 0.3234 | Yes |
| 29 | MTSS1 |  |  | 1046 | 0.205 | 0.3310 | Yes |
| 30 | MTMR9 |  |  | 1055 | 0.204 | 0.3380 | Yes |
| 31 | SLC35F1 |  |  | 1092 | 0.199 | 0.3417 | Yes |
| 32 | ZNF300 |  |  | 1095 | 0.199 | 0.3491 | Yes |
| 33 | REV3L |  |  | 1140 | 0.193 | 0.3516 | Yes |
| 34 | PCSK1N |  |  | 1162 | 0.190 | 0.3566 | Yes |
| 35 | GPT2 |  |  | 1166 | 0.189 | 0.3636 | Yes |
| 36 | KIAA0895L |  |  | 1236 | 0.181 | 0.3628 | Yes |
| 37 | FAM171A2 |  |  | 1280 | 0.174 | 0.3648 | Yes |
| 38 | DNM3 |  |  | 1294 | 0.172 | 0.3700 | Yes |
| 39 | ACVR2B |  |  | 1298 | 0.172 | 0.3763 | Yes |
| 40 | VPS8 |  |  | 1346 | 0.167 | 0.3774 | Yes |
| 41 | RERE |  |  | 1357 | 0.165 | 0.3827 | Yes |
| 42 | SPPL3 |  |  | 1358 | 0.165 | 0.3891 | Yes |
| 43 | EZH1 |  |  | 1394 | 0.162 | 0.3915 | Yes |
| 44 | BMP7 |  |  | 1419 | 0.160 | 0.3950 | Yes |
| 45 | KIF5C |  |  | 1422 | 0.160 | 0.4009 | Yes |
| 46 | HIP1R |  |  | 1446 | 0.157 | 0.4044 | Yes |
| 47 | ABCC5 |  |  | 1456 | 0.157 | 0.4095 | Yes |
| 48 | ZNF248 |  |  | 1459 | 0.157 | 0.4153 | Yes |
| 49 | ARHGEF9 |  |  | 1463 | 0.156 | 0.4211 | Yes |
| 50 | POU2F1 |  |  | 1467 | 0.156 | 0.4268 | Yes |
| 51 | CSMD2 |  |  | 1488 | 0.154 | 0.4305 | Yes |
| 52 | CLASP2 |  |  | 1504 | 0.152 | 0.4347 | Yes |
| 53 | GRK4 |  |  | 1514 | 0.151 | 0.4395 | Yes |
| 54 | SPAST |  |  | 1606 | 0.141 | 0.4347 | Yes |
| 55 | CASK |  |  | 1617 | 0.140 | 0.4390 | Yes |
| 56 | SGSM2 |  |  | 1618 | 0.140 | 0.4445 | Yes |
| 57 | CPT1C |  |  | 1620 | 0.140 | 0.4498 | Yes |
| 58 | GABBR1 |  |  | 1652 | 0.138 | 0.4516 | Yes |
| 59 | SEC61A2 |  |  | 1684 | 0.135 | 0.4533 | Yes |
| 60 | DCLK2 |  |  | 1694 | 0.134 | 0.4575 | Yes |
| 61 | C20orf194 |  |  | 1714 | 0.132 | 0.4605 | Yes |
| 62 | ZNF462 |  |  | 1814 | 0.126 | 0.4542 | No |
| 63 | HMBOX1 |  |  | 1909 | 0.118 | 0.4481 | No |
| 64 | MMP15 |  |  | 1942 | 0.116 | 0.4490 | No |
| 65 | RALGPS2 |  |  | 1955 | 0.115 | 0.4521 | No |
| 66 | PDE10A |  |  | 1993 | 0.112 | 0.4522 | No |
| 67 | CLN8 |  |  | 2056 | 0.108 | 0.4494 | No |
| 68 | DAAM1 |  |  | 2069 | 0.107 | 0.4522 | No |
| 69 | NF1 |  |  | 2105 | 0.105 | 0.4523 | No |
| 70 | LMBR1L |  |  | 2127 | 0.104 | 0.4540 | No |
| 71 | ARVCF |  |  | 2250 | 0.095 | 0.4438 | No |
| 72 | SSH2 |  |  | 2357 | 0.088 | 0.4352 | No |
| 73 | ATF7IP |  |  | 2439 | 0.083 | 0.4293 | No |
| 74 | RNF144A |  |  | 2480 | 0.081 | 0.4279 | No |
| 75 | CPEB4 |  |  | 2511 | 0.078 | 0.4275 | No |
| 76 | NIN |  |  | 2601 | 0.074 | 0.4203 | No |
| 77 | GADD45G |  |  | 2682 | 0.069 | 0.4139 | No |
| 78 | ZSWIM6 |  |  | 2685 | 0.069 | 0.4164 | No |
| 79 | DCC |  |  | 2725 | 0.067 | 0.4146 | No |
| 80 | RASAL2 |  |  | 2733 | 0.066 | 0.4163 | No |
| 81 | ARHGAP33 |  |  | 2767 | 0.064 | 0.4151 | No |
| 82 | PTPRD |  |  | 2784 | 0.063 | 0.4158 | No |
| 83 | MAPK8IP3 |  |  | 2793 | 0.063 | 0.4173 | No |
| 84 | PHYHIPL |  |  | 2821 | 0.062 | 0.4166 | No |
| 85 | NAV1 |  |  | 2828 | 0.061 | 0.4183 | No |
| 86 | PPP1R12B |  |  | 2884 | 0.058 | 0.4144 | No |
| 87 | USP24 |  |  | 2907 | 0.057 | 0.4141 | No |
| 88 | PRKAB2 |  |  | 2946 | 0.055 | 0.4119 | No |
| 89 | BRWD3 |  |  | 3010 | 0.051 | 0.4068 | No |
| 90 | EPHB2 |  |  | 3015 | 0.051 | 0.4083 | No |
| 91 | ABAT |  |  | 3020 | 0.051 | 0.4098 | No |
| 92 | ZNF711 |  |  | 3086 | 0.048 | 0.4043 | No |
| 93 | WWC1 |  |  | 3113 | 0.046 | 0.4032 | No |
| 94 | NLGN2 |  |  | 3127 | 0.046 | 0.4035 | No |
| 95 | C2orf72 |  |  | 3156 | 0.044 | 0.4020 | No |
| 96 | KIF1A |  |  | 3320 | 0.035 | 0.3849 | No |
| 97 | KIF3A |  |  | 3442 | 0.030 | 0.3723 | No |
| 98 | MED13L |  |  | 3482 | 0.028 | 0.3690 | No |
| 99 | KLHL24 |  |  | 3502 | 0.027 | 0.3679 | No |
| 100 | EVL |  |  | 3508 | 0.027 | 0.3684 | No |
| 101 | TNIK |  |  | 3539 | 0.025 | 0.3660 | No |
| 102 | CA10 |  |  | 3556 | 0.025 | 0.3651 | No |
| 103 | NCOA2 |  |  | 3634 | 0.022 | 0.3573 | No |
| 104 | DAB1 |  |  | 3774 | 0.016 | 0.3422 | No |
| 105 | DCLK1 |  |  | 3776 | 0.016 | 0.3427 | No |
| 106 | FHOD3 |  |  | 3802 | 0.016 | 0.3405 | No |
| 107 | TSTD2 |  |  | 3824 | 0.015 | 0.3387 | No |
| 108 | ZNF532 |  |  | 3873 | 0.013 | 0.3337 | No |
| 109 | MBTD1 |  |  | 3875 | 0.013 | 0.3341 | No |
| 110 | SBK1 |  |  | 3926 | 0.010 | 0.3288 | No |
| 111 | MAPT |  |  | 4051 | 0.005 | 0.3150 | No |
| 112 | EEPD1 |  |  | 4100 | 0.003 | 0.3097 | No |
| 113 | MIIP |  |  | 4340 | -0.007 | 0.2829 | No |
| 114 | ZMIZ1 |  |  | 4395 | -0.010 | 0.2772 | No |
| 115 | SATB1 |  |  | 4505 | -0.014 | 0.2654 | No |
| 116 | ATG2B |  |  | 4515 | -0.015 | 0.2649 | No |
| 117 | C5orf30 |  |  | 4578 | -0.017 | 0.2586 | No |
| 118 | FOXN3 |  |  | 4639 | -0.020 | 0.2525 | No |
| 119 | SHC3 |  |  | 4768 | -0.025 | 0.2390 | No |
| 120 | FBXL16 |  |  | 4780 | -0.025 | 0.2387 | No |
| 121 | CDO1 |  |  | 4786 | -0.025 | 0.2391 | No |
| 122 | GSTA4 |  |  | 5179 | -0.039 | 0.1963 | No |
| 123 | RBPJ |  |  | 5474 | -0.051 | 0.1649 | No |
| 124 | PHLPP1 |  |  | 5506 | -0.053 | 0.1635 | No |
| 125 | KIF26B |  |  | 5534 | -0.054 | 0.1626 | No |
| 126 | FAM107B |  |  | 5545 | -0.055 | 0.1635 | No |
| 127 | ATCAY |  |  | 5683 | -0.061 | 0.1504 | No |
| 128 | PJA1 |  |  | 5763 | -0.064 | 0.1439 | No |
| 129 | TMOD2 |  |  | 5775 | -0.065 | 0.1452 | No |
| 130 | FAM19A5 |  |  | 5803 | -0.066 | 0.1447 | No |
| 131 | WASF1 |  |  | 6054 | -0.077 | 0.1194 | No |
| 132 | MAPRE2 |  |  | 6079 | -0.078 | 0.1197 | No |
| 133 | AKT3 |  |  | 6191 | -0.084 | 0.1104 | No |
| 134 | YLPM1 |  |  | 6277 | -0.089 | 0.1042 | No |
| 135 | HIPK2 |  |  | 6320 | -0.091 | 0.1030 | No |
| 136 | RPAIN |  |  | 6664 | -0.112 | 0.0685 | No |
| 137 | CCDC28B |  |  | 6753 | -0.118 | 0.0631 | No |
| 138 | NUDT11 |  |  | 6758 | -0.118 | 0.0672 | No |
| 139 | GNG2 |  |  | 6841 | -0.124 | 0.0627 | No |
| 140 | CLIP3 |  |  | 6897 | -0.129 | 0.0615 | No |
| 141 | GSK3B |  |  | 6951 | -0.133 | 0.0607 | No |
| 142 | SMAP2 |  |  | 6983 | -0.135 | 0.0624 | No |
| 143 | KIF2A |  |  | 7030 | -0.139 | 0.0626 | No |
| 144 | TSHZ1 |  |  | 7088 | -0.144 | 0.0618 | No |
| 145 | RAP2A |  |  | 7134 | -0.148 | 0.0624 | No |
| 146 | CRMP1 |  |  | 7368 | -0.177 | 0.0429 | No |
| 147 | CELF2 |  |  | 7440 | -0.185 | 0.0421 | No |
| 148 | UBL3 |  |  | 7525 | -0.194 | 0.0401 | No |
| 149 | CACNG4 |  |  | 7712 | -0.220 | 0.0276 | No |
| 150 | SOX4 |  |  | 7958 | -0.268 | 0.0102 | No |
| 151 | TMSB15A |  |  | 8046 | -0.284 | 0.0114 | No |
| 152 | RAB2A |  |  | 8273 | -0.346 | -0.0008 | No |
| 153 | ENHO |  |  | 8432 | -0.400 | -0.0031 | No |
| 154 | PFN2 |  |  | 8545 | -0.455 | 0.0019 | No |
| 155 | PEG10 |  |  | 8628 | -0.510 | 0.0124 | No |
| 156 | MARCKSL1 |  |  | 8857 | -0.716 | 0.0144 | No |
Table: GSEA details [plain text format]

  

Fig 2: GAROFANO\_IAVARONE\_NEU\_SUBTYPE: Random ES distribution      
 Gene set null distribution of ES for **GAROFANO\_IAVARONE\_NEU\_SUBTYPE**
